# Inhibition of miR-19a protects neurons against ischemic stroke through modulating glucose metabolism and neuronal apoptosis

**DOI:** 10.1186/s11658-019-0160-2

**Published:** 2019-05-31

**Authors:** Xiao-Li Ge, Jin-Li Wang, Xin Liu, Jia Zhang, Chang Liu, Li Guo

**Affiliations:** 1 Department of Neurology, The Second Hospital of Hebei Medical University, Shijiazhuang, 050000 China; 2 Department of Neurosurgery, The Second Hospital of Hebei Medical University, Shijiazhuang, 050000 China; 3 Department of Obstetrics, The Second Hospital of Hebei Medical University, Shijiazhuang, 050000 China; 4 Department of Rehabilitation, The Second Hospital of Hebei Medical University, Shijiazhuang, 050000 China

**Keywords:** Ischemic stroke, miR-19a-3p, ADIPOR2, Glucose metabolism, Apoptosis

## Abstract

**Background:**

Accumulating evidence has shown that altered microRNA (miR) modulation is implicated in the pathologies of ischemic stroke. However, it is unclear whether and how hsa-miR-19a-3p mediates cerebral ischemic injury. Herein, we investigated the functional role of miR-19a-3p in cerebral ischemic injury and explored its underlying regulatory mechanism.

**Methods:**

In vivo ischemic/reperfusion (I/R) neuronal injury and in vitro oxygen-glucose deprivation (OGD) were established. Expression of miR-19a-3p was determined by quantitative real-time polymerase chain reaction (qRT-PCR). Glucose uptake, lactate production, and apoptosis were determined. *ADIPOR2* was predicted as a target of miR-19a-3p in silico and experimentally validated by qRT-PCR, Western blot analysis and luciferase assay assays.

**Results:**

MiR-19a expression was significantly downregulated and upregulated in rat neurons and astrocytes, respectively (*P* < 0.01). A significantly elevated level of miR-19a-3p was found in I/R and OGD models in comparison to sham/control groups (P < 0.01). Expression of the glycolysis enzyme markers LDHA, PKM2, HK2, Glut1 and PDK1, apoptosis-related factors levels, apoptosis, glucose uptake, and lactate production were significantly repressed by both I/R and OGD (P < 0.01 in each case). Moreover, miR-19a-3p mimic aggravated, while miR-19a-3p inhibitor alleviated, the above observations. *Adipor2* was predicted and confirmed to be a direct target of miR-19a. Furthermore, restoration of *Adipor2* reversed miR-19a-3p-induced effects.

**Conclusions:**

Collectively, our results indicate that elevated miR-19a-3p mediates cerebral ischemic injury by targeting ADIPOR2. MiR-19a-3p attenuation thus might offer hope of a novel therapeutic target for ischemic stroke injury treatment.

## Introduction

Brain ischemic stroke is known as the commonest cerebrovascular disease and a primary public health problem, characterized by a high incidence and mortality, acute onset, rapid development and severe outcomes, and unfortunately, its incidence is on the rise [1–3]. Cerebral ischemic injury, identified as the fundamental pathophysiological basis of ischemic stroke, has a complicated pathogenesis concerning multiple biological events, such as neuronal apoptosis [4, 5], astrocyte activation [6, 7], proinflammatory reaction and oxidative stress [8, 9]. Cerebral ischemia results in brain impairment on account of deprivation of oxygen and glucose resulting from blockage of local blood supply. In spite of substantial advances in cerebral ischemic injury therapeutic strategies, the therapeutic effect is still far from ideal for a large number of patients, which is possibly due to a lack of clear understanding of the pathological process [10–12]. Thereby, delineating the molecular mechanism of ischemic stroke is urgent to develop effective therapies for cerebral ischemic injury.

MicroRNAs (miRs), 21–22 nucleotides long, are short noncoding RNAs and are known to function in post-transcriptional regulation of gene expression through majorly binding the 3′-untranslated region (UTR) of their target messenger RNA (mRNA), accordingly modulating cells’ behaviors [13, 14]. The broadly conserved miR-19a-3p, a crucial component of the miR-17-92 cluster, has been shown to be involved in pathogenesis/disease progression in lung cancer [15], gastric cancer [16], breast cancer [17], and hepatocellular carcinoma [18]. Furthermore, miR-19a-3p has been found to be overexpressed in glioma cells and astrocytic glioma tissues and its overexpression is strongly linked with malignancy grades in glioma patients, indicating an important role in gliomagenesis [19]. MiR-19a-3p is increased and positively correlated with poor survival in astrocytoma patients [20]. MiR-19a-3p has been shown to stimulate axonal outgrowth in embryonic cortical neurons [21]. Importantly, the miR-17-92 cluster is upregulated in neural progenitor cells of mouse, and its overexpression observed either in cultured ischemic neural progenitor cells or in the subventricular zone of ischemic animals markedly accelerated cell proliferation, while repression of individual components of the miR-17-92 cluster, miR-18a and miR-19a-3p, inhibited cell proliferation and enhanced cell death [22]. However, these studies have largely been correlative and the role of miR-19a-3p in cerebral ischemic injury has not been investigated; hence it was investigated in I/R and OGD models in the current study.

## Materials and methods

### Isolation and culture of neuronal cells and astrocytes

Primary neurons were isolated from newborn Sprague Dawley (SD) rats. First, the hippocampal tissues were digested by 0.125% trypsin for 10 min. After separation and centrifugation, the obtained neurons were inoculated into 6-well plates after the wells were treated by poly-d-lysine (Sigma-Aldrich, St. Louis, MO, USA) for 12 h. The cells were maintained using neurobasal medium containing with 2% B27, 1% glutamine and cytarabine at 37 °C with 5% CO_2_. To establish the oxygen-glucose deprivation (OGD) model, neurons were cultured in deoxygenated, glucose-free Hanks’ Balanced Salt Solution at 37 °C with 5% CO_2_ and 95% N_2_ for 24 h. Then the cells were cultured under normoxic conditions.

Primary astrocytes were isolated from newborn SD rats. The neocortices were treated using 0.09% trypsin for 25 min after they were dissected. The single cells obtained after centrifugation were inoculated into 6-well plates treated by poly-d-lysine (Sigma-Aldrich). Eagle’s Minimal Essential Medium was used to culture cortical astrocytes with 10% FBS, 21 mM glucose and 10% EGF (Gibco, Grand Island, NY, USA). Cells were cultured at 37 °C with 5% CO_2_.

### Rat ischemic stroke model establishment

**Fig. 1:**
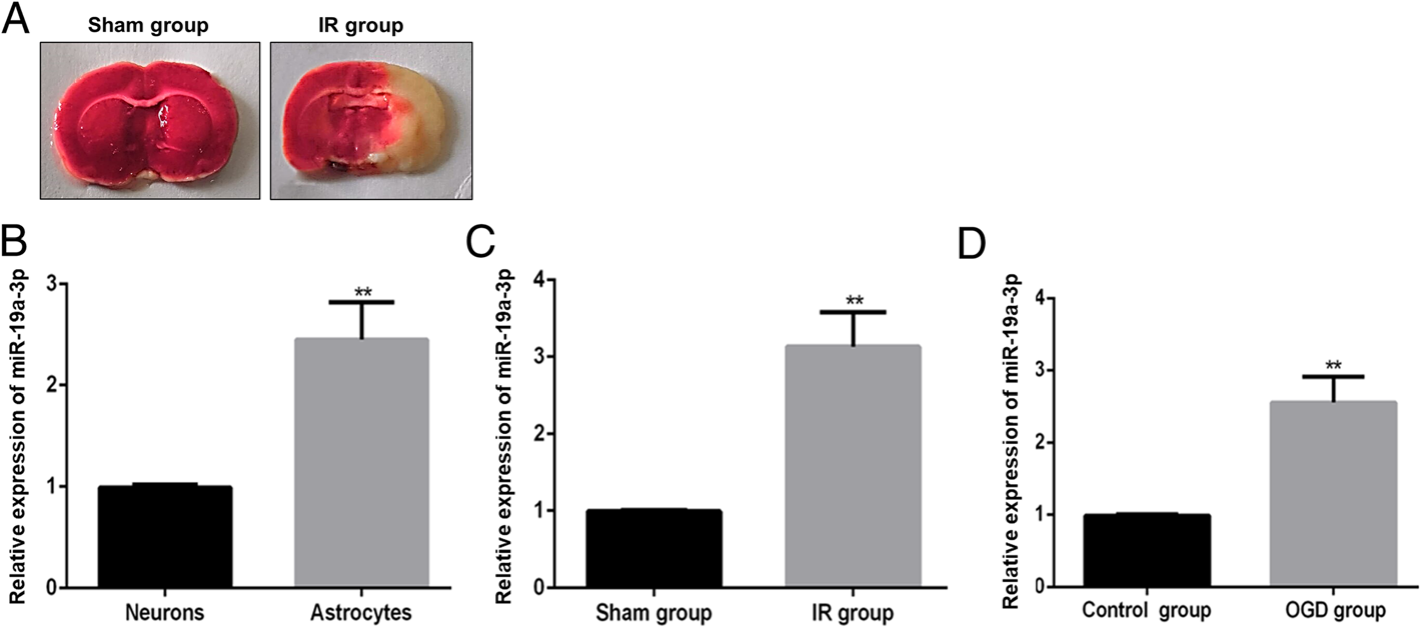
Level of miR-19a-3p in astrocytes or neurons, and IR/OGD models. **a**, 2,3,5-triphenyltetrazolium chloride (TTC) staining of IR and sham groups to confirm successful ischemia induction. Representative images are shown. **b**, Difference in the expression level of miR-19a-3p in astrocytes and neurons. **c**, Difference in the expression level of miR-19a-3p in IR (astrocytes) model compared with sham group. **d**, Difference in the expression level of miR-19a-3p in OGD (neurons) model compared with control group. Results are representative of 3 independent biological replicates. IR and sham are from 5 animals each (**P < 0.01)

The Ethics Committee of the Second Hospital of Hebei Medical University has reviewed and approved this study. All the experimental procedures were carried out according to the United States National Institutes of Health guidelines for the use of experimental animals. Rat ischemic stroke models were established by performing middle cerebral artery occlusion in adult (8–10 weeks) male Sprague Dawley (SD) rats weighing 200 ± 12 g. All rats were anesthetized using sodium pentobarbital with a concentration of 100 mg/kg. Cervical incision was performed to expose arteries. The right internal carotid artery and middle cerebral artery were ligated by a nylon suture for 90 min. Then all rats were recovered for 24 h before being sacrificed. 2,3,5-triphenyltetrazolium chloride (TTC) staining was performed using routine protocols to confirm successful ischemia induction (Fig. 1a). Ischemia induction was confirmed by a blinded pathologist by reviewing the TTC staining of the experimental groups.

### Quantitative real-time polymerase chain reaction (qRT-PCR)

Total RNA was extracted using Trizol (Invitrogen, Carlsbad, California, USA). To generate cDNA, the Mir-X miRNA FirstStrand Synthesis Kit and the Reverse Transcriptase Kit (Takara, Dalian, China) were used. The qPCR was carried out using a SYBR Premix Ex Taq TM II (Takara) with the ABI 7900 qRT-PCR System (Applied Biosystems, Foster City, CA, USA). The primer sequences used were as follows: mature rno-miR-19a-3p stem-loop PCR (product size: 71 bp): F: 5′-TGTGCAAATCTATGCAAA-3′, R: 5′-CAGTGCGTGTCGTGGAGT-3′; U6: F: 5′-CGCAAGGATGACACGCAAAT-3′, R: 5′-ATTTGCGTGTCATCCTTGCG-3′; *Adipor2* (product size: 140 bp): F: 5′-GGAGTGTTCGTGGGCTTGGG-3′, R: 5′- GCAGCTCCTGTGATATAGAGG-3′; *Gapdh* (product size: 177 bp): F: 5′- ATGACATCAAGAAGGTGGTG-3′; R: 5′-CATACCAGGAAATGAGCTTG-3′. U6 and *Gapdh* were used for normalization of miR-19a-3p and *Adipor2* expression. Data were analyzed with the 2^-ΔΔCt^ method and expressed as folds over experimental control groups.

### Western blot

Total proteins were isolated using RIPA lysis Buffer (Beyotime, Shanghai, China). Protein concentrations were tested using a bicinchoninic acid (BCA) kit (Beyotime). Twenty μg of proteins were resolved by sodium dodecyl sulphate-polyacrylamide gel electrophoresis (SDS-PAGE). Membranes were probed with primary antibodies against LDHA (1: 1000, Abcam, Cambridge, USA), PKM2 (1: 1000, Abcam), HK2 (1: 1000, Abcam), Glut1 (1: 1000, Abcam), PDK1 (1: 1000, Abcam), Bim (1: 1000, Abcam), Bax (1: 1000, Abcam), caspase-9 (1: 500, Abcam), ADIPOR2 (1: 1000, Abcam), and GAPDH (1: 1000, Beyotime). GAPDH was used as the loading control protein. Densitometry analyses was performed using Image J software.

### Transfection

Cells (5 × 10^5^ cells/well) were transfected with miR-19a-3p mimic or inhibitor of miR-19a-3p (Shanghai GenePharma Co., Ltd., Shanghai, China), and their controls using Lipofectamine 2000 (ThermoFisher Scientific, Shanghai, China). Cells were harvested after 48 h of transfection to perform follow-up experiments as indicated.

### Luciferase assay

The potential targets of miR-19a-3p were in silico predicted using TargetScan software. The wild or mutant (miR-19a-3p seed deleted (596–603)) of the 3′-UTR of *Adipor2* was cloned into the pmiRGLO vector (Promega, Madison, WI, USA). The miR-19a-3p mimic and its control were transfected into neuronal cells. After 48 h of transfection, the relative luciferase activities of cells were measured by the Dual-Glo Luciferase Assay System (Promega) in accordance with the manufacturer’s introductions. Renilla luciferase activity was used to normalize luciferase activity.

### Terminal-deoxynucleotidyl transferase-mediated UTP nick end labeling (TUNEL) staining

Cell apoptosis was tested by TUNEL staining using the One Step TUNEL Apoptosis Assay Kit (Beyotime) following the manufacturer’s guidelines. After TUNEL staining, cells were incubated with DAPI. Cells were imaged using a fluorescence microscope (Fluoview FV1000, Olympus, Tokyo, Japan) and counted under five random fields. Neuronal apoptosis was assessed by the percentage of TUNEL-positive cells from the total number of cells (DAPI- positive cells).

### Glucose metabolism assays

For determining the change of glucose metabolism, the uptake of glucose and production of lactate analyses were assessed by the Glucose Uptake Colorimetric Assay Kit and Lactate Colorimetric Assay Kit (Sigma-Aldrich, St Louis, MO, USA) following the manufacturer’s protocol.

### Statistical analysis

Statistical analysis was conducted using SPSS Statistics software 22.0 (Chicago, IL, USA). Data are represented (except otherwise mentioned) as mean ± standard deviation (SD). The differences between two groups were analyzed using Student’s *t*-test. The differences of multiple groups were performed by one-way ANOVA followed by post-hoc Dunnett t test. *P* < 0.05 was considered as statistically significant.

## Results

### Low expression of miR-19a-3p in rat neurons and miR-19a-3p upregulation in I/R and OGD models

We initially determined whether basal miR-19a-3p expression was different in neuronal cells and astrocytes, two important components affected in ischemic brain injury. MiR-19a-3p level was significantly lower in neurons compared to astrocytes (Fig. 1b; **P < 0.01). However, induction of I/R in vivo in astrocytes or OGD in vitro in neuronal cells significantly induced miR-19a-3p expression compared to the sham or control group, respectively, and it was up-regulated in I/R and OGD models compared with sham/control groups (Fig. 1c and d, **P < 0.01), highlighting that miR-19a-3p might either be involved in or serve as an effector of ischemic brain injury.

### Glucose metabolism is repressed by IR/OGD treatment

**Fig. 2:**
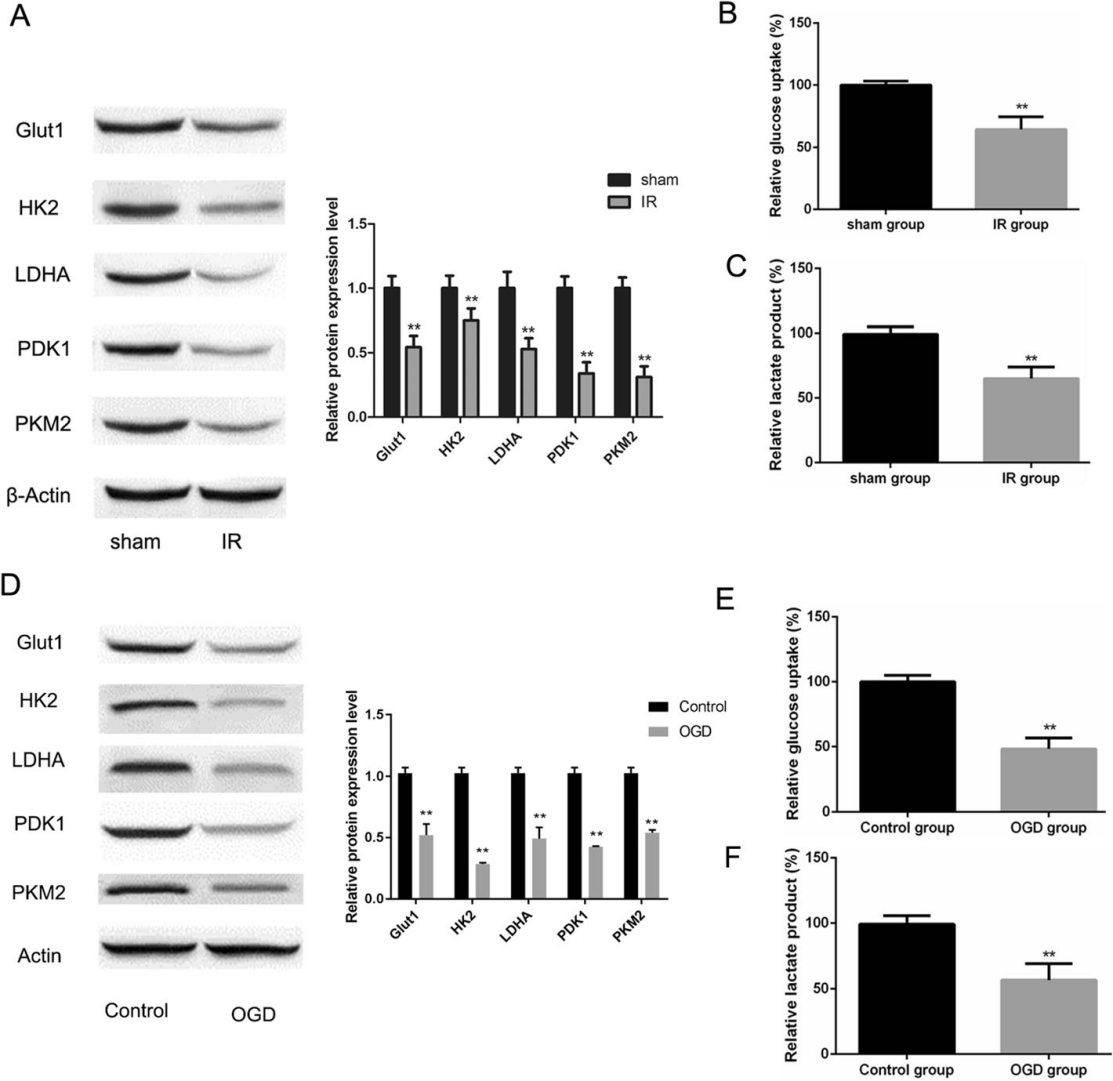
Impaired glucose metabolism after IR/OGD. **a**, **d** The expression levels of glycolytic enzymes, LDHA, PKM2, HK2, Glut1 and PDK1 in IR and sham groups (**a**) and control and OGD groups (**d**) were analyzed by western blot. Representative images and densitometric analysis of blots are shown. **b**, **d** Change in glucose uptake in IR and sham groups (**b**), and control and OGD groups (**e**). **c**, **f** Difference of lactate production in IR/sham (**c**) and OGD/control groups (**f**). For **a**-**c**, results represent 5 animals per group. For **d**-**f**, results represent 3 biological replicates. Data in **b**, **c**, **e**, and **f** were expressed as means ± SD. (**P < 0.01 vs. control group)

It is well known that ischemic brain injury results in deprivation of cellular energy reserve leading to death in cells. We thus assessed expression levels of glycolysis enzyme-associated factors LDHA, PKM2, HK2, Glut1 and PDK1, glucose uptake and lactate production after IR/OGD. LDHA, PKM2, HK2, Glut1 and PDK1 expression (Fig. 2a, d), glucose uptake (Fig. 2b, e) and lactate production (Fig. 2c, f) were significantly decreased in astrocytes within the IR and neuronal cells within the OGD group (**P < 0.01 in each case). These results indicate that glucose metabolism rescue aids in ameliorating ischemic brain injury.

### Neuronal apoptosis is induced by OGD treatment

**Fig. 3:**
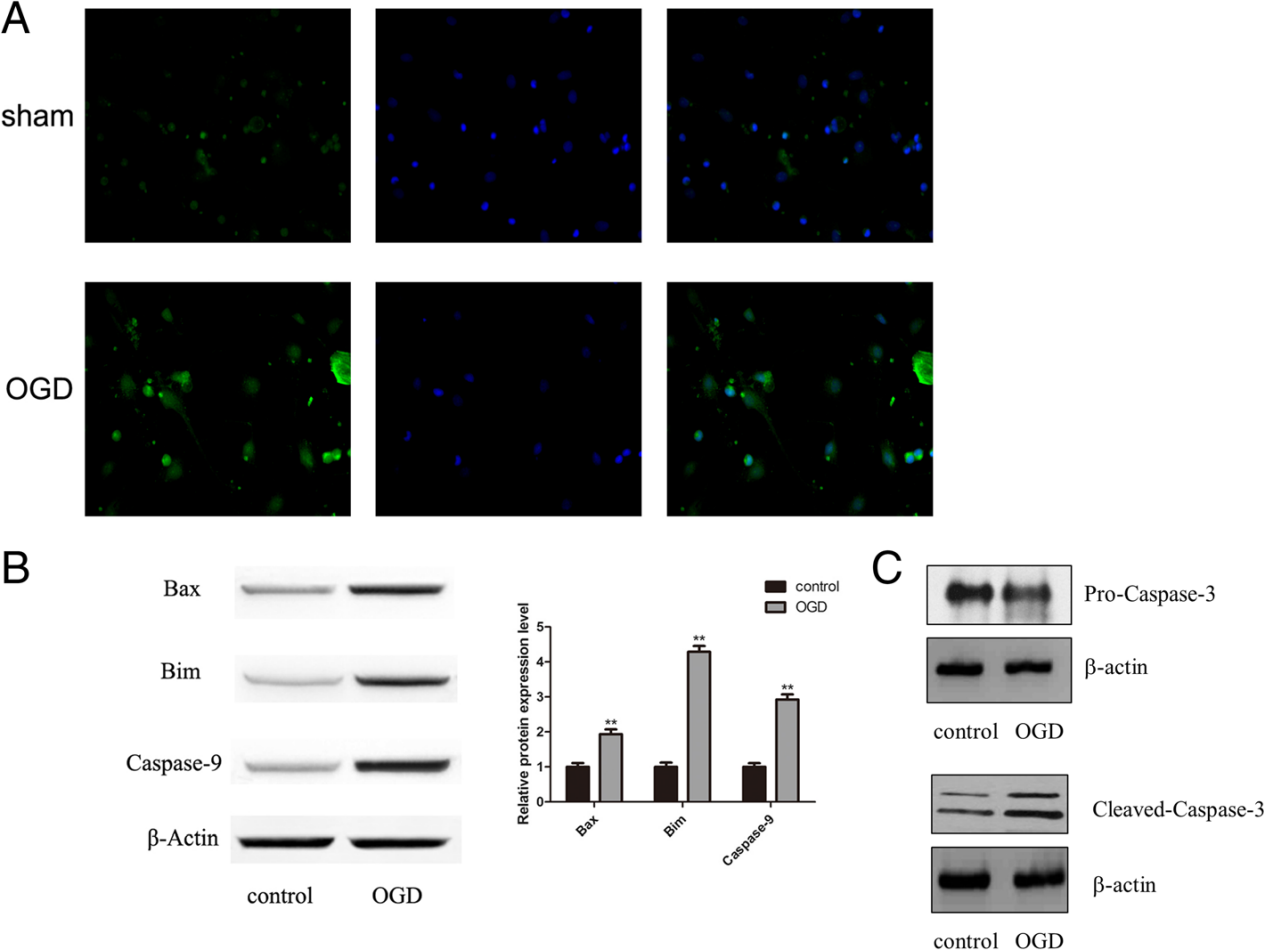
OGD-induced neuronal apoptosis. **a**, TUNEL staining of cells in control and OGD groups. Data are representative of three independent experiments. **b**, The expression levels of pro-apoptotic proteins Bax, Bim and caspase-9 were analyzed by western blot. **c**, The expression levels of pro-apoptotic proteins, pro-caspase-3 and cleaved-caspase-3 were analyzed by western blot. Representative blots of three independent experiments are shown in **b** and **c**. Densitometry analysis was expressed as means ± SD. (**P < 0.01 vs. control group)

For detecting neuronal apoptosis induced by OGD, apoptosis and levels of apoptosis-related proteins were measured using TUNEL and western blot analyses, respectively. TUNEL-stained cells were significantly more numerous in the OGD group compared to the control group (Fig. 3a; **P < 0.01). Concomitantly, the apoptosis markers Bax, Bim and caspase-9 were detectably upregulated in OGD neurons, but not in controls (Fig. 3b; **P < 0.01). Expression of pro- and cleaved-caspase-3 confirmed increased apoptosis in the OGD treatment groups (Fig. 3c).

### MiR-19a inhibits glucose uptake and promotes neuronal apoptosis by targeting *Adipor2*

**Fig. 4:**
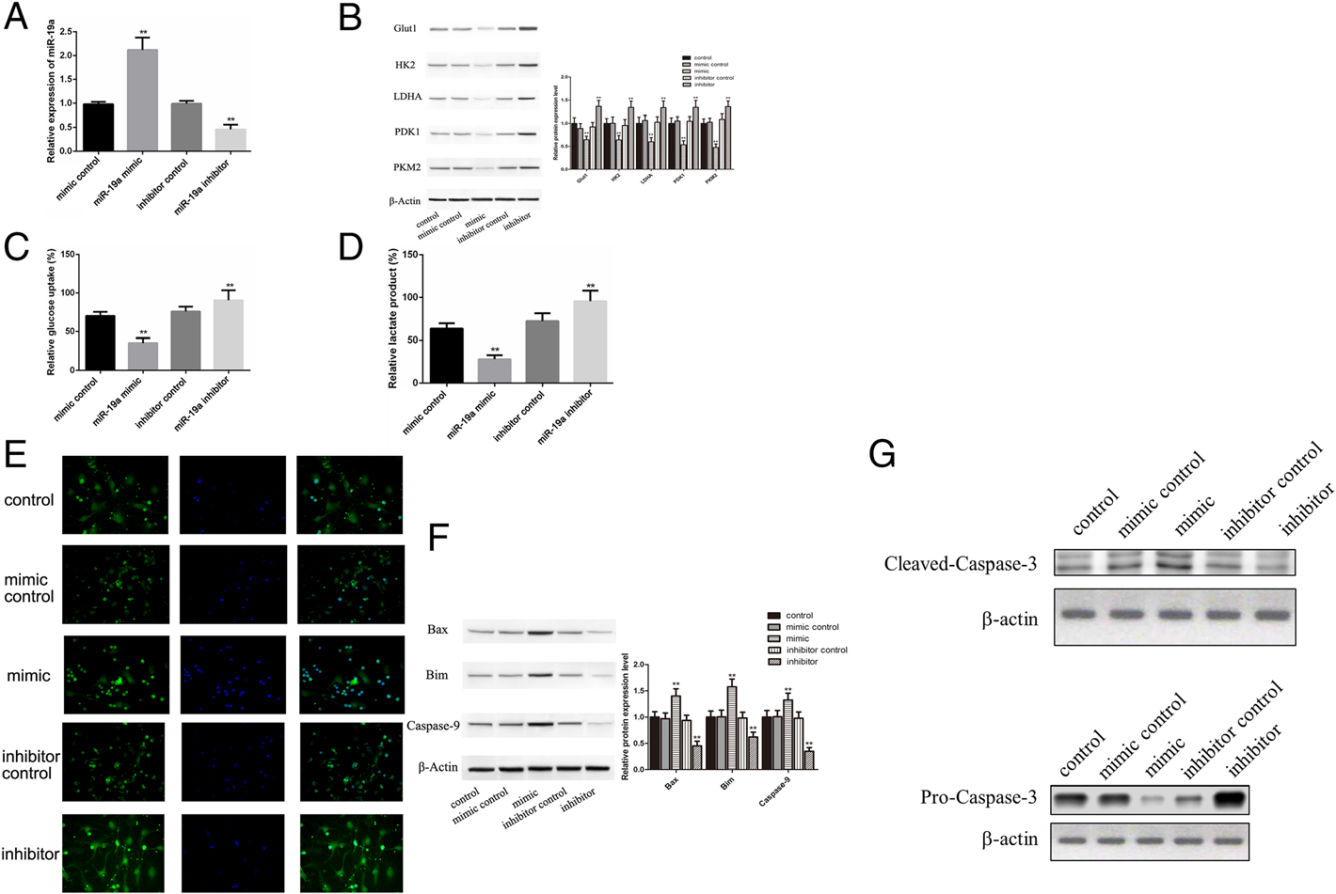
Effect of miR-19a mimic/inhibitor on glucose metabolism and apoptosis in OGD-treated neurons. **a**, Successful transfection of miR-19a-3p mimic/inhibitor was verified by qRT-PCR. B-F, The effects of miR-19a-3p on expression of glycolytic enzymes (**b**), glucose uptake, (**c**) lactate production (**d**), and apoptosis (**e**-**g**) were assayed as described in Figs. 2 and 3. All data were expressed as means ± SD. (**P < 0.01 vs. control group)

**Fig. 5:**
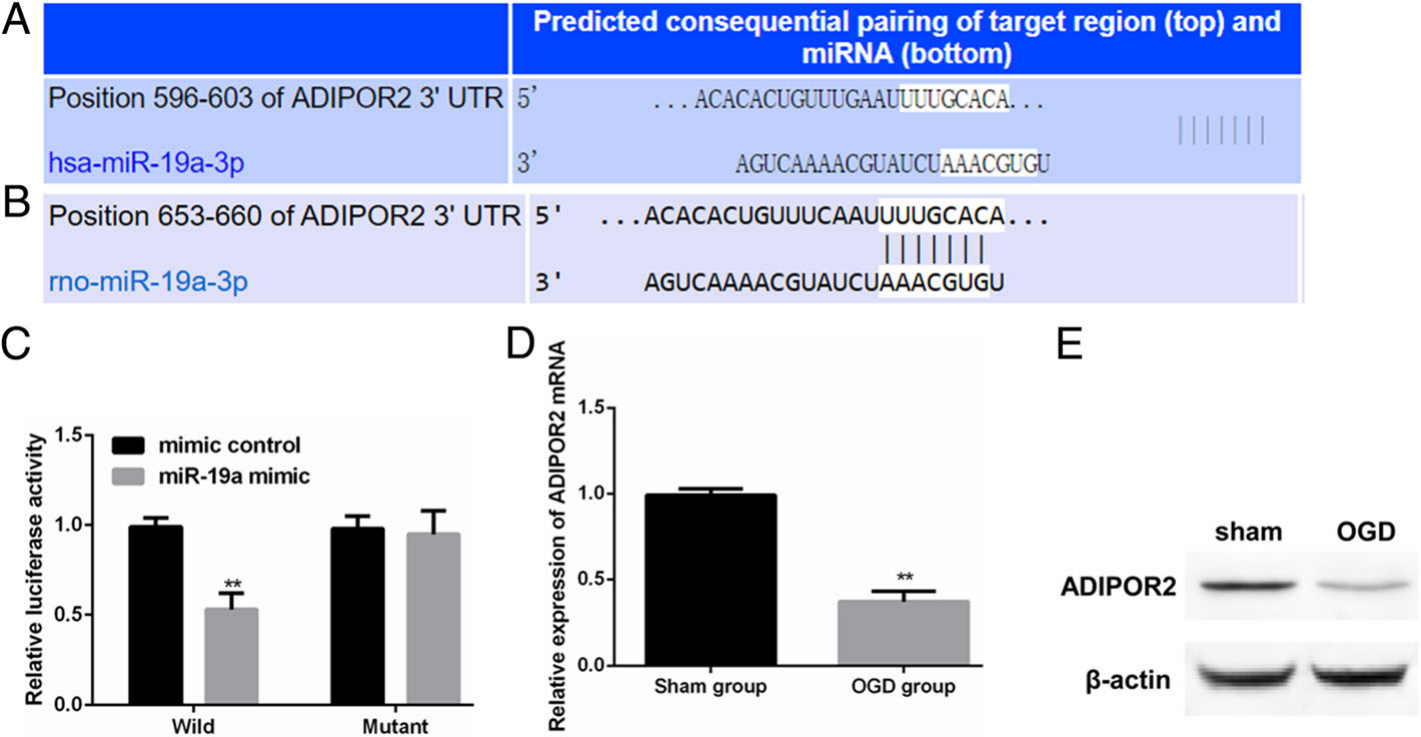
*Adipor2* is a bona fide target of miR-19a-3p. **a**, **b** Predicted (using TargetScan) target site of miR-19a-3p in the 3′-UTRs of human (**a**) and rat (**b**) *ADIPO2* mRNA. **c**, Relative activities of wild-type and mutant (miR-190-3p binding site deletion mutant) luciferase reporters in neurons transfected with miRNA-19a-3p mimic or control. Data represent mean ± standard deviation of three independent experiments. **d**, **e**, *Adipor2* mRNA (**d**) and protein (**e**) expression in OGD and control groups. All data are representative of three independent experiments and were expressed as means ± SD. (**P < 0.01 vs. control group)

To determine the linkage between miR-19a-3p expression and glucose metabolism or neuronal apoptosis in OGD, miR-19a-3p mimic or inhibitor was transfected to neuronal cells in the OGD and control groups. Successful miR-19a-3p overexpression and knockdown by the mimic and inhibitor, respectively, was confirmed (Fig. 4a, **P < 0.01). Expression of glycolysis enzymes, glucose uptake and lactate production were markedly reduced after miR-19a-3p mimic transfection and were rescued when upregulated in OGD neurons (Fig. 4b-d; **P < 0.01). Inhibition of miR-19a-3p by the inhibitor downregulated both apoptosis (Fig. 4e) and expression of pro-apoptosis markers (Fig. 4f-g),

To investigate downstream targets of miR-19a-3p that might be responsible for its role in ischemic injury, we used TargetScan software to predict putative targets. There were more than 500 putative miR-19a-3p targets predicted. One of the predicted targets, *Adipor-2* (encoding for adiponectin receptor 2), was of particular interest for two reasons. First, adiponectin receptors have been shown to be expressed in specific neurogenic niches and associated with brain repair [23]. Secondly, *Adipor-1* has been shown to mediate electroacupuncture mediated cerebral attenuation of cerebral I/R injury in diabetic mice [24]. Hence, we proceeded to determine whether *Adipor2* is a direct target of miR-19a-3p and one of the genes post-transcriptionally regulated by miR-19a-3p during cerebral I/R injury. TargetScan predicted that miR-19a-3p targets position 596–603 of human *ADIPOR2* and position 653–660 of the rat *Adipor2* 3′-UTR, which were entirely conserved in the sequence (Fig. 5a, b). Luciferase expression of wild-type *Adipor2* 3′-UTR harboring Firefly luciferase ORF was significantly downregulated following transfection of miR-19a-3p mimic (Fig. 5b; **P < 0.01). However, luciferase expression of the reporter was not changed following miR-19a-3p transfection in neuronal cells when the miR-19a-3p seed was mutated in the reporter (Fig. 5b), confirming *Adipor2* as a bona fide target of miR-19a-3p. We next investigated whether increased miR-19a-3p in OGD neurons actually results in downregulation of *Adipor2*. Both *Adipor2* mRNA (Fig. 5c) and protein expression (Fig. 5d) was significantly downregulated in OGD neurons compared to the control group. Cumulatively, these results indicated that increased miR-19a-3p in the OGD neuronal cells is targeting and downregulating expression of *Adipor2*.

### ADIPOR2 is the functional effector of miR-19a-3p during cerebral ischemic injury

**Fig. 6:**
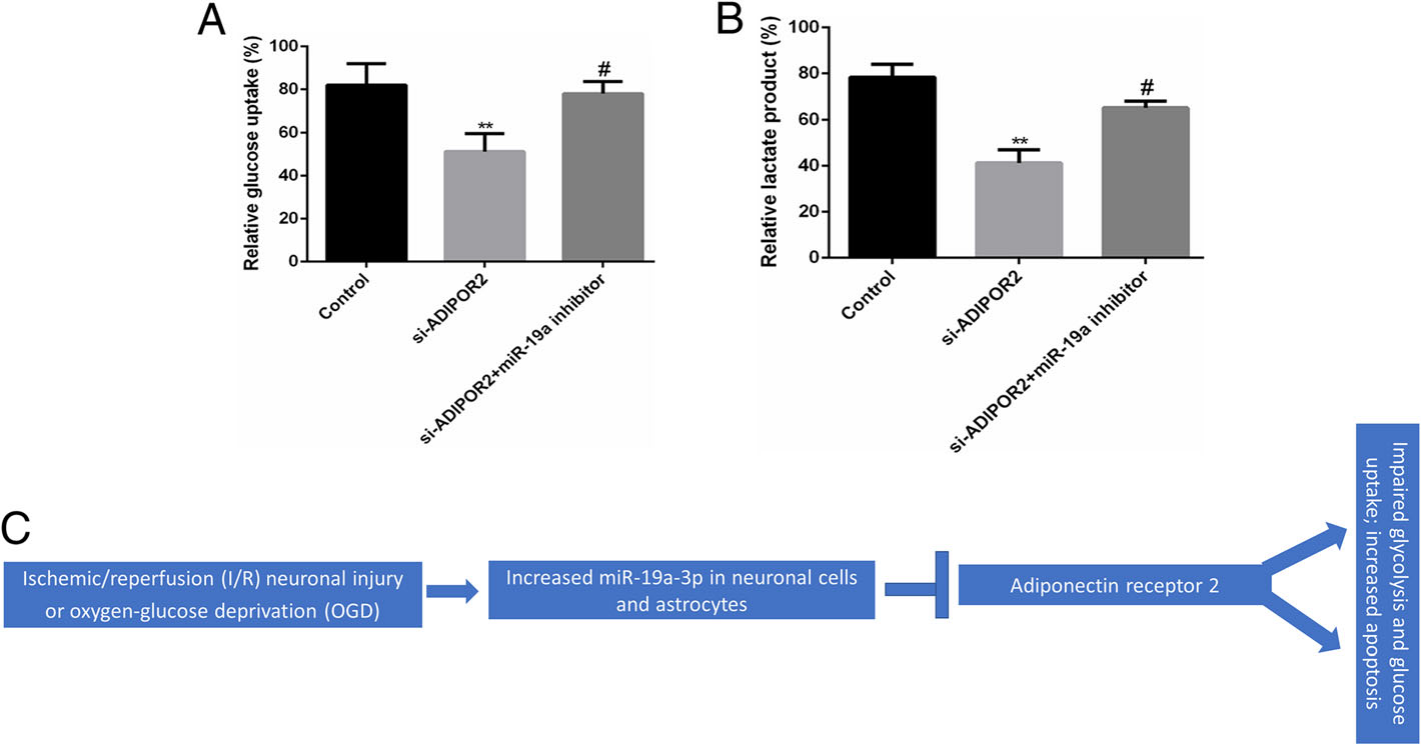
*Adipor2* is an effector of miR-19a-3p’s role in inducing cerebral ischemic injury. **a**, **b**, Glucose uptake (**a**) and lactate production (**b**) in neuronal cells transfected with siRNA-targeting *Adipor2* either alone or in combination with anti-miR-19a-3p inhibitor. All data are representative of three independent experiments and were expressed as means ± SD. (**P < 0.01 vs. control group, #*P* < 0.05 vs. si- ADIPOR2 group). **c**, Model summarizing the major findings of the current study highlighting how elevated miR-19a-3p expression during IR and OGD can result in impaired glucose metabolism and apoptosis by targeting *Adipor2*

Even though our results confirmed that *Adipor2* is a bona fide target of miR-19a-3p, it still did not imply that the pathogenic effect of miR-19a-3p during I/R injury is being mediated by ADIPOR2. To ascertain this, we transfected OGD neurons with siRNA targeting *Adipor2*, either alone or along with the miR-19a-3p inhibitor. *Adipor2* silencing in control neurons downregulated glucose uptake and lactate production, while co-transfection with the miR-19a inhibitor mitigated these phenomena (Fig. 6a, b), confirming that the ischemic brain damage mediated by miR-19a-3p is mediated at least in part by downregulation of *Adipor2*.

## Discussion

In the present study, we observed that miR-19a-3p was higher in I/R and OGD models than controls. Significantly, miR-19a-3p inhibition effectively mitigated IR/OGD-induced repression of glycolysis enzymes’ expression, glucose uptake and lactate production, and neuronal apoptosis, which was regulated by targeting *Adipor2* (Fig. 6c). Collectively, our observations elucidate one target gene which is downregulated by elevated miR-19a-3p during cerebral ischemic injury pathogenesis.

Astrocytes, identified as the largest number of cells in the central nervous system (CNS), interact with neurons in order to maintain the stability of the CNS environment [25–27]. In response to cerebral ischemia, astrocytes undergo hyperplasia and metabolic reserve enhancement and delayed apoptosis is observed in neurons [28–30]. Aberrant miRNA expression levels have been correlated with models of cerebral ischemia injury [31–33]. MiR-19a-3p has been found to highly correlate with the development of a variety of cancers, especially in astrocytoma [20]. Overexpression of miR-19a-3p is observed in astrocytoma patients; in addition, its overexpression occurred in cultured ischemic neural progenitor cells and promoted cell proliferation [22], implying that miR-19a-3p plays a role in regulating neural cell function. One study emphasized the cell-type-specific expression of miR-19a-3p by detecting high expression in astrocytes and a low level in neurons, suggesting a probable impaired effect of miR-19a-3p in preserving neural function.

During cerebral ischemia, nerve cells undergo irreversible damage due to the complete cessation of glucose and oxygen supply to the ischemic core [34–37]. Thus, ameliorating glucose metabolism and controlling neural cell apoptosis is of great importance to improve cerebral ischemia damage [38, 39]. Modulation of miR-19a-3p expression impacted glycolytic enzymes and neuronal apoptosis, even though our results do not indicate the specific downstream effectors that are directly regulating these processes.

*ADIPOR2*, encoding adiponectin receptor2, has been implicated in cardiovascular diseases [40, 41], fatty liver disease [42], diabetes [43], diabetic nephropathy [44], and bone metabolic disorders [45]. In each of these pathogenic conditions forced overexpression of ADIPOR2 ameliorates the disease process. A correlation between ADIPOR2 expression and ischemic stroke has also been previously established [46]. Our experiments corroborate these findings and highlight the potential need to test whether adenovirus-mediated overexpression of ADIPOR2 will be a viable therapeutic intervention in in vitro and in vivo models of cerebral ischemic injury. In light of recent reports emerging that offer contradictory findings about the dual role of astrocyte and astrocyte-neuron interactions in metabolic alterations accompanied by cerebral ischemia brain injury, further work will be required to investigate the significance of astrocytes. Additionally, the role of other targets of miR-19a-3p need to be evaluated.

## Abbreviations

BCA: Bicinchoninic acid
CNS: Central nervous system
ECL: Enhanced chemiluminescence
HRP: Horseradish peroxidase
miRs: MicroRNAs
OGD: Oxygen-glucose deprivation model
SD: Sprague Dawley
SD: standard deviation
SDS-PAGE: Sodium dodecyl sulphate-polyacrylamide gel electrophoresis
TUNEL: Transferase-mediated UTP nick end labeling
UTR: 3′-untranslated region
